# CMR evaluation of aorto-pulmonary collaterals in Glenn and Fontan patients

**DOI:** 10.1186/1532-429X-17-S1-Q101

**Published:** 2015-02-03

**Authors:** Pierluigi Festa, Lamia Ait Ali, Petra Keilberg, Vitali Pak

**Affiliations:** 1 Pediatric cardiology and GUCH unit, Toscany Fondation Gabriel Monasterio, Massa, Italy; 2 MRI Lab, FTGM, Pisa, Italy; 3 Istitut of Clinical Physiology, Massa, Italy; 4 Pediatric cardiac surgery, FTGM, Massa, Italy

## Background

Aorto-pulmonary collaterals (APCs) are frequent in patients with uni-ventricular heart. Their clinical significance remains controversial. Quantitative assessment of APCs blood flow using cardiac magnetic resonance (CMR) have been already validated.

Aim: to evaluate factors associated to APCs flow (QAPCs) evaluated by CMR in Post Glenn and Fontan patients

## Methods

Form our CMR database we identified all patients with previous Glenn or Fontan intervention who underwent a targeted CMR studies from May 2005 to September 2014. QAPCs was calculated using through-plane phase-contrast as QAPCs = (left pulmonary veins flow + right pulmonary veins flow) - (right pulmonary artery flow + left pulmonary artery flow). Values were normalized to body surface area and indexed to aortic flow (QAPCs/aortic flow). Surgical history and clinical status was recorded.

## Results

86 CMR examination in 82 patients (55 Fontan and 31 Glenn, age 14 ±11) were included in the study. In 4 patients CMR evaluation was performed before and after Fontan.

QAPCs resulted 1.06 ± 0.7 l/min/m^2^, constituting 29 ± 19 % (range 3-88%) of systemic blood flow. The amount of QAPCs in Glenn patients resulted higher than in Fontan patients (respectively 1.26±0.58 l/min/m^2^ vs 0 9±0.7 l/min/m2 , p=0.03). The QAPCs was independent of the morphology of the systemic ventricle and the anatomical diagnosis except for hypoplastic left heart syndrome in the Glenn group (1.7±0.38 l/min/m^2^ vs 1.13±0.58 l/min/m^2^ in all other patients, p=0.02). In the whole population, QAPCs inversely correlates with age at CMR study (r= -0.21, P= 0.001), with peripheral 02 sat (r= -0,38, P<0.001), with Right Pulmonary Artery (RPA) diameter, cross sectional area and flow (respectively r= -0.18 P=0,03, r=-0.15, P=0,02, r=-0.4, P<0,001), as well as with Left Pulmonary Artery (LPA) diameters (r=-0.43, P<0.001), cross sectional area (r=-0.41, P< 0.001) and flow (r=-0.49, P<0.001); QAPCs directly correlates with cardiac output (r=0.51 P<0,001). We didn't found any correlation between QAPCs and age at Glenn or Fontan operation in both groups, ventricular volumes and EF. In the 4 patients evaluated before and after Fontan the QAPCs decreased.

## Conclusions

New CMR techniques allow a reliable quantification of APCs flow. It is higher in Glenn patients than in Fontan and is inversely correlated to O2 sat, to pulmonary branches diameters and flow and directly correlate to the cardiac output. HLHS is a risk factor for APCs. Prospective study are needed to evaluate the clinical significance of APCs

## Funding

N/A.

